# Visual Functions Generating Conscious Seeing

**DOI:** 10.3389/fpsyg.2020.00083

**Published:** 2020-02-14

**Authors:** Victor A. F. Lamme

**Affiliations:** Department of Psychology, Amsterdam Brain and Cognition (ABC), University of Amsterdam, Amsterdam, Netherlands

**Keywords:** consciousness, neural correlate of consciousness, global neuronal workspace theory, integrated information theory, higher order thought theory, recurrent processing theory, perceptual organization

## Abstract

Visual functions are reviewed that coincide with conscious as opposed to unconscious vision. Four stages of vision are identified, going from the fully invisible, to subjectively invisible, unattended, and clearly visible. It is proposed that feature extraction, categorization, and some aspects of visual inference occur during full and subjective invisibility. Functions related to perceptual organization, such as grouping and figure-ground segregation, occur during inattention as well as full visibility. It is argued that perceptual organization is the function that is central to understanding the transition from unconscious to conscious seeing. It is discussed what this implies for theories of consciousness such as Recurrent Processing Theory, Higher Order Thought Theory, Integrated Information Theory, and Global Neuronal Workspace Theory.

## Where Does Conscious Vision Start, Where Does It End?

What does it mean to consciously see or not see? The matter is simple at the extreme ends. Suppose someone is shown a face, which is then recognized by the subject, who insists that he was seeing that face and can describe all sorts of features. Then it will be safe to assume there was a conscious sensation of that stimulus. At the other end, suppose someone is shown visual stimuli (or blanks) that are strongly masked, while all attention is focused on the screen, and the only task is to press one button when seen, and another when not seen. If the subject then insists not having seen anything at any trial, which is confirmed by *d*-prime being 0 (and sufficient trials were recorded to reliably estimate that), it will be safe to assume that there was no conscious sensation of the stimuli. This is generally considered “objective invisibility,” the level at which conscious sensations are trusted to be absent.

But with anything in between, matters immediately get murky. There is currently much debate about what should count as evidence for unconscious vision. Objective invisibility may be considered too “harsh” a criterion. Another way to gauge the transition from unconscious to conscious perception is the “perceptual awareness scale” (PAS), where subjects report the amount of subjective awareness on a scale, say from 1 to 4 (Ramsøy and Overgaard, 2004). The scale typically has labels describing different qualitative sensations a stimulus may evoke, such as “not seeing anything,” “a weak glimpse,” “almost clear image” up to “a clear image,” that can also be tailored to specific stimulus details. Depending on these descriptions, and on how subjects interpret them, different thresholds are found for stimuli that are masked, degraded or otherwise reduced in visibility. Often (if not always), stimuli that are above the level of objective invisibility and hence have *d*-prime above 0 may get rated “unaware” (level 1 on the PAS) in such subjective scaling experiments (Wierzchoń et al., 2014), implying that subjective invisibility may happen for stimuli that are objectively detectable.

Other variations on this theme exist. Some have combined objective measurements with confidence ratings or post-decision wagering, asking subjects how much they trust their yes-no responses. When confidence is equally high (or low) for correct and incorrect trials (i.e., metacognition about one’s own performance is absent), stimuli are considered metacognitively unconscious. A big advantage of this approach is that objective and subjective (or rather metacognitive) visibility can be directly compared in the framework of signal detection theory, using a d-prime value (Fleming and Lau, 2014), and hence solving some issues of decision criteria that the PAS may suffer from. Metacognitive visibility is typically reached at stimulus strengths that are above objective invisibility, yet are lower than or equal to those for subjective awareness as measured with a PAS (Jachs et al., 2015).

**Figure 1:**
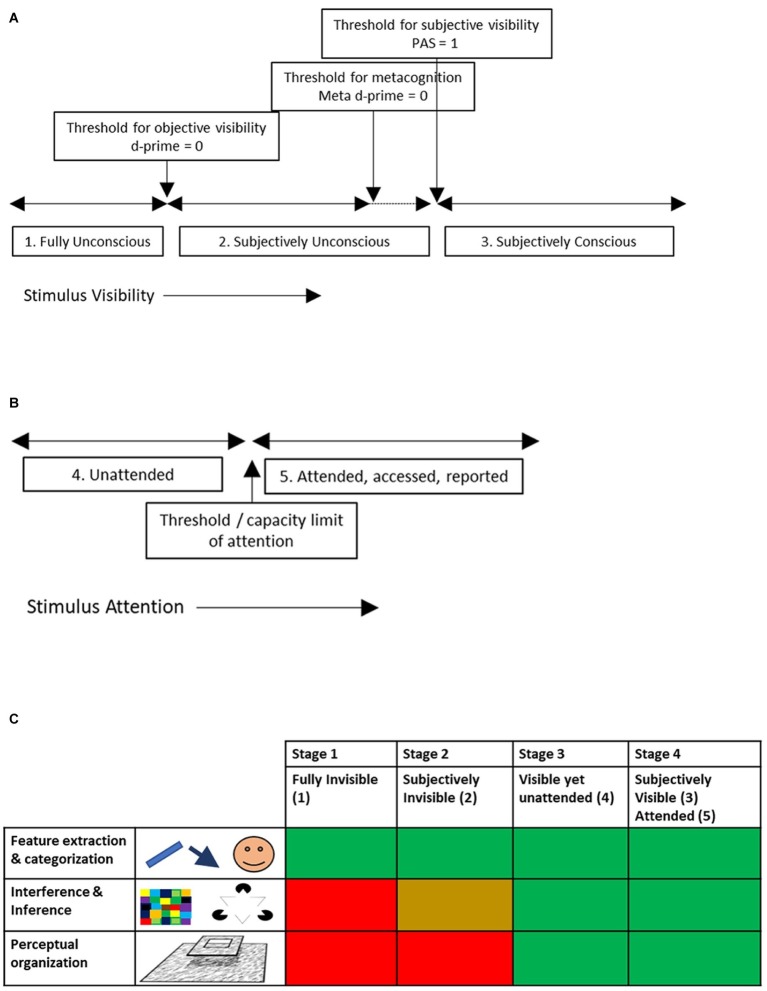
Visual operations during stages of unconscious to conscious and attentive (visual) processing. **(A)** Depending on stimulus manipulations like masking, CFS, etc., stimuli may be consciously visible or not. Shown are the different thresholds for conscious visibility, depending on the method used to “ask” subjects whether the stimulus was seen or not. **(B)** Depending on attention, stimuli may become consciously accessed and reported or not. There is typically a capacity limit for how much can be attended, accessed or reported. **(C)** Table showing which of three categories of visual functions are or are not executed during the different stages of unconscious/conscious/unattended/attended vision. A red box indicates that the functions are not executed, green that they are, yellow that there are mixed results or that some functions in this category are executed while others are not.

From this, we arrive at three stages of visual processing (Figure 1A):

1. Fully unconscious, i.e., stimuli that are below the threshold for objective visibility.
2. Subjectively unconscious, i.e., stimuli that are reported as unseen in experiments using perceptual awareness scales, meta d-prime, confidence ratings etc., yet are above the threshold for objective invisibility.
3. Subjectively conscious, i.e., stimuli that get a PAS score above 1, where meta-d prime is above 0, etc., and hence are reported as seen.

On another dimension are phenomena of “unconscious” vision that relate to attention. Well-known are the change blindness (CB) or innatentional blindness (IB) demonstrations where huge changes between scenes are not seen, or gorillas pass by unnoticed. Often used is the attentional blink (AB) paradigm, where the reporting of a first target renders a shortly following second target “invisible.”

A separate distinction thus is between (Figure 1B):

4. Unattended, i.e., stimuli that are reported as not seen while attention is diverted away from them (as in CB, IB or AB experiments). These are potentially visible, yet momentarily escape cognitive access.
5. Attended, accessed, reported, memorized, etc.

How to compare these stages? Stages 3 and 5 are in fact similar, since in both cases we have subjects reporting (and most likely having attention for and access to) subjectively visible stimuli. It is less clear to what extent “unattended” overlaps with subjective invisibility. Stimuli that are subjectively easily visible become subjectively invisible when attention is diverted away (which is in fact the striking point of the change blindness phenomenon). When attention happens to be directed at these stimuli, the subjective invisibility is resolved. This is clearly different from invisibility from masking, flash suppression, etc. In those cases, there is nothing the subject himself can do to see the stimulus, it seems irrevocably “buried” in the unconscious. It thus seems that inattentional invisibility is a more “cognitive” or higher-level form of invisibility than invisibility from masking, flash suppression, etc. As will be shown below, indeed attention as manipulated in CB, IB or AB paradigms does not interfere with visual functions per se. It therefore seems warranted to categorize “unattended” as “potentially visible,” yet not reportable or accessed right now, as others have done before (Dehaene et al., 2006).

A potential “hierarchy” of visual phenomenology thus may be (above levels in parentheses)

1. Stage 1: Fully invisible (1)
2. Stage 2: Subjectively invisible (2)
3. Stage 3: Unattended (but otherwise subjectively visible) (4)
4. Stage 4: Subjectively visible and attended (3/5)

An alternative is to view the two types of manipulations of conscious vision – those manipulating visibility and those manipulating attention- as orthogonal [as I and others have done before (Lamme, 2003, 2004; Dehaene et al., 2006)]. In fact, it is irrelevant for the discussion below (about which visual functions operate under the four levels) whether the four levels are orthogonal or hierarchical.

Another debate is where the “real” boundary between conscious and unconscious vision lies. Some have doubts as to whether “subjectively invisible” (stage 2) is truly unconscious, for example because subjective awareness may be influenced by all sorts of non-visual conditions, like the type of PAS scale used, whether it is followed or preceded by other questions (Overgaard and Sandberg, 2012), decision criteria, etc. Another discussion is whether the absence of attention (stage 3) really implies the absence of conscious perception (rather than the absence of attention, access and report *of* that conscious percept). That has been discussed in previous work extensively, resulting in an apparent stalemate of opinions about the essence of conscious vision (Koch et al., 2016; Lamme, 2018) (although in my view some arguments can be made to lay the boundary between stages 2 and 3 rather than 3 and 4 (Lamme, 2010), supporting the somewhat counterintuitive view that “invisibility” due to inattention is in fact not true invisibility, yet a case of failure to “internally report” or otherwise cognitively access and manipulate the information. Further arguments and experiments may pull us out of this complex discussion). The controversy also has links to much older philosophical debates about the so-called hard problem, and the distinction between phenomenal (P-) and access (A-) consciousness (Block, 2005). I will refrain from any reiteration of these debates and simply try to link each level to various functions.

For further clarity, I will assume that:

1. Anesthesia renders everything stage 1.
2. Manipulations like backward or dichoptic masking, CFS, and rivalry, or conditions like blindsight render stimuli stage 1 when *d*-prime is really 0 (which is not often achieved), otherwise stage 2.
3. Attentional manipulations like AB, IB, and CB, or attentional deficits like neglect speak only to the transition between stages 3 and 4 (see above for the underlying reasoning).

## What Cognitive Functions to Consider?

The four stages identified obviously – and tri*via*lly – relate to several cognitive functions. All measurements of consciousness in one way or another rely on subjects reporting, mostly *via* button presses. The required motor machinery consists of a whole set of cognitive functions that are not relevant to the discussion here. Metacognition and attention are trivially linked to conscious vision, simply because they are part of the way the different stages are distinguished. Metacognition is intertwined with various aspects of decision making. Closely associated with attention is a function called “access,” or the making available of sensory information to other cognitive processes (Naccache, 2018). These are all functions that come with the “definition” of conscious perception that is used, and a discussion of their role in consciousness amounts to nothing more than a reiteration of the stalemate previously mentioned (although interesting observations about the relation between attention, metacognition, access and consciousness have been made).

Furthermore, I will here limit myself to visual functions only. What will be obtained is a set of functions that coincide with conscious perception, which is more or less the classic idea behind the search for the neural correlate of consciousness (NCC) (Koch et al., 2016). A potential next step then is to argue for these functions to also be both necessary and sufficient for the “machinery” that is “generating” the conscious percept: some functions (such as orientation selectivity in V1) contribute to vision, just like the eye is required to see, but they do not by themselves generate a conscious percept (just like an isolated eyeball will not), while other functions necessarily coincide with the occurrence of conscious percepts; without them, no conscious percept, with them, the percept follows automatically. The logic behind the following is that visual functions that occur without subjects having conscious sensations are not sufficient for generating conscious vision. Functions that only occur when conscious percepts are present are candidate NCC’s, and this paper is an attempt to identify those functions. Note, however, that the definition of what exactly entails a true NCC is complex (Owen and Guta, 2019).

## Visual Operations During Stage 1 and Beyond: Feature Extraction and Categorization

Everything retinal seems to proceed regardless of consciousness; most of what we know of retinal processing comes from studying isolated retinas or anesthetized animals. So the feature extraction that occurs there (basically converting a luminance distribution into a distribution of contrast and its temporal dynamics) is generally considered to be independent of consciousness. Similarly, the transmittance of retinal signals to the cortex *via* the LGN seems unaffected by it (Durand et al., 2016). The extraction of features like orientation, direction of motion, disparity, or color in early visual cortex, as well as higher level categorizations of for example complex shapes or even faces in higher visual regions occurs just as well during anesthesia, masking, rivalry, or inattention as during conscious vision. In fact, any visual operation that is “hardwired” *via* feedforward connections does not depend on any form of consciousness. This also includes so called “base groupings” (Roelfsema, 2006) such as the co-occurrence of a particular orientation with a particular direction of motion, as many cells are simultaneously selective for both features. In sum, everything there is to “know” in terms of localized features and categories about the visual scene is extracted [during a “feedforward sweep” through the brain (Lamme and Roelfsema, 2000)] regardless of consciousness.

## Visual Operations During Stage 2 and Beyond: Interference and Inference (To Some Extent)

At some point in vision, all the features and categories that have been extracted start to interact. Some examples of these are phenomena of perceptual interference, where the luminance or color of one part of the image is influenced by that of another, potentially resulting in striking visual illusions of brightness or color differences between physically identical patches of gray or color. The neural correlates of such interactions are found in many visual areas, and to some extent also in anesthetized animals (Rossi et al., 1996; Shapley and Hawken, 2011). Moreover, they seem present under conditions of masking or CFS, albeit not with the strictest criteria of invisibility (Harris et al., 2011). An important test case is the phenomenon of color constancy, the ability to interpret color from the pattern of wavelengths across the visual scene. Some experiments suggest this to occur already during stage 2 (Stoerig and Cowey, 1989), others do not (Breitmeyer et al., 2004; Barbur and Spang, 2008).

A related visual function is that of inference. These are most poignantly illustrated by many visual illusions. Take the well-known Kanizsa triangle, where the physical stimulus consists of three “Pac-men” shapes. From that configuration we infer a triangle overlying three disks, with clearly visible yet illusory contours, and even an increased brightness for the triangle region. Neural correlates of such illusory contours have been found in early visual cortex, in awake animals. Some have shown these illusions to persist during CFS (Wang et al., 2012), others claim they do not (Harris et al., 2011). Blindsight patients report Kanizsa illusions when one of the inducers is in the blind hemifield (Marcel, 1998), suggesting they are generated partly unconsciously.

In sum, it seems that the visual processes of interference and inference, where local features start to interact and put into global perceptual context (e.g., wavelength being converted to color, contours to surfaces, etc.) find their basis in stage 2, and may even operate to completion during this stage. Some have called this stage the formation of the 2.5D sketch (Nakayama and Shimojo, 1992), i.e., going from features to surfaces and their relative positions.

**Figure 2:**
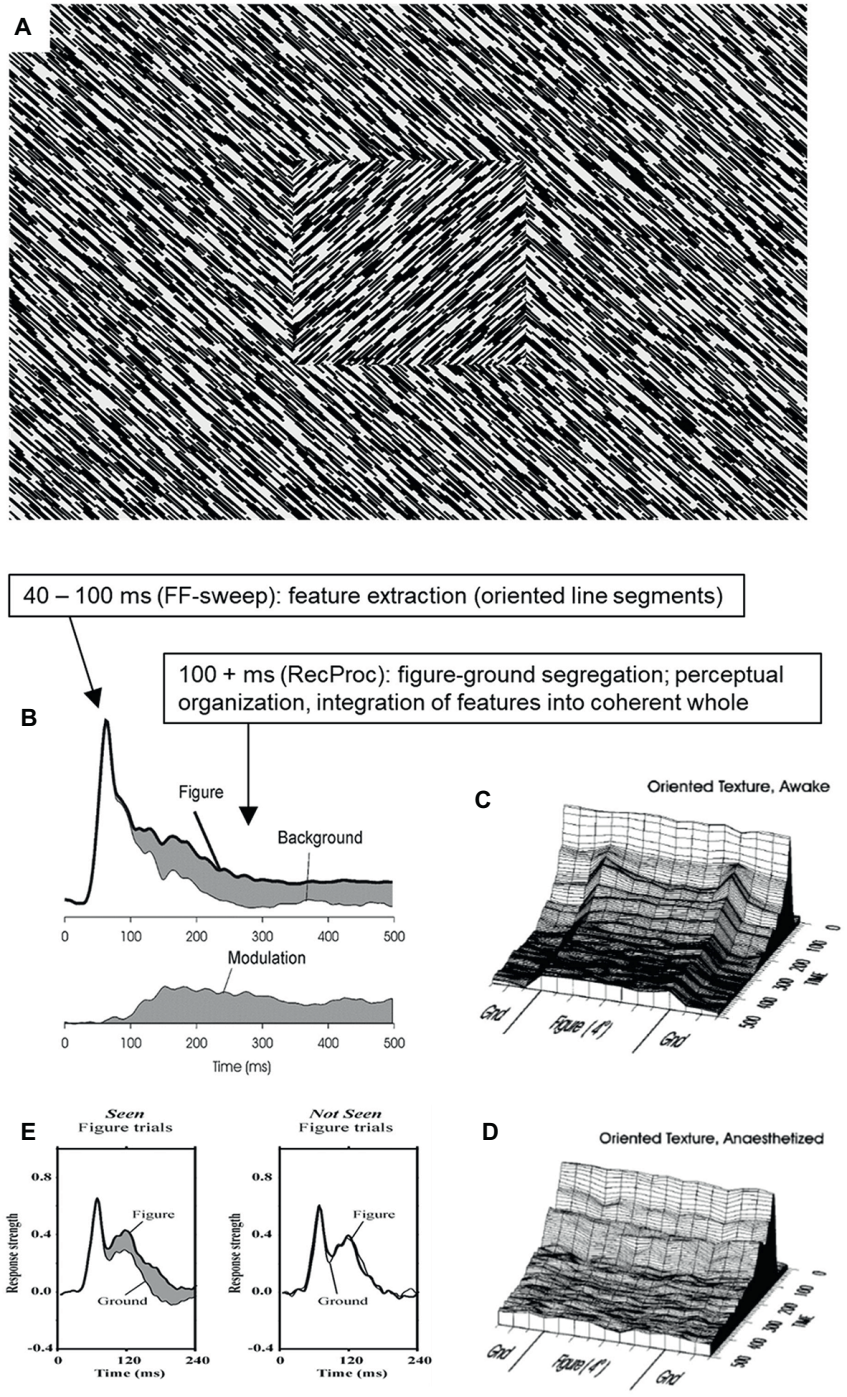
The neural processing of figure ground organization and its relation to conscious perception. **(A)** A typical figure-ground stimulus as used in many studies (of the author) on the relation between perceptual organization and conscious vision. **(B)** Typical result from recording responses in monkey primary visual cortex (V1) to the stimulus shown in **(A)**. Note how the initial response is identical for line segments belonging either to figure or background, yet that later responses reflect the processing of figure versus background (Lamme, 1995; Zipser et al., 1996). **(C)** Contextual modulation reflects figure-ground segregation as modulation is uniformly elevated for the figure region (Lamme et al., 1999). **(D)** When anesthetized, feedforward activation and detection of line segments is still present, yet the figure-ground modulation is selectively absent (Lamme et al., 1998). **(E)** When figures are not seen, feedforward V1 responses persist, but figure-ground modulation is selectively absent (Supèr et al., 2001). © (1998) National Academy of Sciences.

There are clear limits, however, to what is achieved in terms of moving toward a more global interpretation of the scene during stage 2. An important aspect of scene interpretation is perceptual organization, where distant features are combined into coherent objects and their relative layout. An example is figure ground organization as depicted in Figure 2A. The scene is interpreted as a textured square figure overlying a textured background, a percept entirely based on the grouping of similarly oriented line segments, segregating the two groups, and assigning the one as figure and the other as background (perceptually “continuing” behind the figure). I have devoted a career to studying the neural mechanisms behind this stimulus (Figures 2B,C), and how it depends on factors like attention (Lamme, 2003), anesthesia (Figure 2D; Lamme et al., 1998), masking (Lamme et al., 2001), lesions (Lamme et al., 1998), pharmacological interventions (Meuwese et al., 2013), and what not. While the processing of the orientation of line segments survives stage 2 manipulations (like masking), the figure-ground percept and its neural correlates do not (Lamme et al., 2001; Fahrenfort et al., 2007). Any neural signal representing the figure-ground layout [also in more complex stimulus configurations than shown here (Zipser et al., 1996)] critically depends on the layout being consciously visible (Figures 2D,E; Lamme and Spekreijse, 2000; Supèr et al., 2001; Lamme, 2004, 2009), and hence is absent during stage 2.

## Visual Operations During Stage 3 and Beyond: Anything Visual Is Completed

Effects of attention on visual processing are well-documented. In general, unattended stimuli generate weaker responses than attended ones. But the quality of visual representation seems to suffer much less, if at all. For example, the width of orientation tuning curves in V1 does not change during inattention, only their amplitude does (McAdams and Maunsell, 1999). Higher level representations, such as selective responses to faces or other categories in the temporal lobe typically get weaker (Kastner and Ungerleider, 2000) but essentially remain (Marois et al., 2004).

Processes of interference and inference proceed as well, as Kanizsa illusion representations are identical whether attended and reported or inattended (Vandenbroucke et al., 2014). Fahrenfort et al. (2017) studied the neural processing of Kanizsa figures using clever control stimuli that enabled a distinction between the processing of the inducers that support the illusion and the processes of integration and inference that “make” the illusion happen. It was found that masking selectively disrupts the neural processing of integration and inference, supporting the limits of stage 1 and 2 processing discussed above. However, when the Kanizsa stimuli were made “invisible” due to an attentional blink manipulation, the inference and integration related neural signals remained. Also, the neural signals related to perceptual organization and figure ground segregation (Figure 2), which are absent in stage 2, are present during stage 3, as was shown in a variety of studies using change detection (Landman et al., 2003) and inattention paradigms (Scholte et al., 2001).

In sum, although attention has strong effects on the strength of visual representations in visual cortex [and may also lower thresholds for otherwise invisible stimuli (Kastner and Ungerleider, 2000)], the essence of what visual cortex does – going from feature extraction and categorization toward perceptual inference, grouping, and organization, so as to “build” a complete interpretation of the visual scene in front of us – is essentially unaffected by attention, and hence completed in stage 3 (Lamme, 2003). Attention has well-documented effects on visual processing, but does not seem to affect whether particular visual functions are executed or not.

## Visual Operations During Stage 4: Cognitive Functions Added

With everything visual completed already during stages 1, 2, and 3, one may wonder what exactly is added in stage 4. The short answer is: nothing in terms of visual operations. What is added are cognitive functions that may operate on the visual representation that is now complete. These may include attentional selection (“highlighting” some parts of the scene, which typically renders their neural representation stronger relative to unattended parts), working memory (storing some parts of the scene), access (making available the visual representation to other functions like control or language), decision processes (deciding on which object to act), metacognition [re-representing the visual representation somewhere else (Brown et al., 2019)], and report (you saying “I saw this”). These may alter the visual representation somewhat, but it should be noted that visual percepts are typically cognitively “impenetrable” (Pylyshyn, 1999). For example, you cannot un-see a visual illusion by knowing it is an illusion.

## What Functions Support the Conscious Percept?

A summary of the results presented above is given in Figure 1C. Note that this is a short review of the matter, and therefore quite simplified, sketching a broad picture. There are exceptions and debatables to the general observations made here (Lamme, 2015). Note that the hierarchy of four stages proposed above (as opposed to the stages being independent or orthogonal) is supported by the findings: invisibility due to attentional manipulations (stage 3) indeed is more “cognitive” or high level, in the sense that it is not caused by a disturbance of any visual process. Invisibility due to manipulations like masking or flash suppression (stage 2) is caused by a disturbance of such visual processes [see (Fahrenfort et al., 2017) for a study manipulating both types of visibility in a single experiment]. The two types of invisibility thus are clearly different “beasts,” which argues for the two not being treated as equal ways of manipulating consciousness (Kim and Blake, 2005).

What does this say about the NCC? As noted, finding the NCC is ultimately aimed at unraveling the set of neural operations (hence functions) that “generate” the conscious (visual) percept. Often, this is framed as operations being both necessary and sufficient for consciousness. In practice, however, this is hard to establish. What mostly occurs is that some correlation is found between conditions or manipulations that affect conscious experience on the one hand, and neural signals and their associated processes on the other hand. On the basis of what is summarized here, some visual functions can be excluded from the NCC in this correlational sense: feature extraction, categorization, some interference and inference occur regardless of whether one is conscious of the visual stimulus or not. Other functions are likely candidates: some inference and integration (like in the Kanizsa illusion), processes of grouping, perceptual organization and figure-ground segregation depend strongly on the stimulus that is evoking these operations being consciously perceived.

Others have argued for the NCC also needing to have some explanatory power toward consciousness. The properties of conscious experience most in need for such explanation according to Seth (2009) are the fact that conscious experiences are (1) simultaneously integrated and differentiated, (2) have some “point of view” or perspective relative to the “I,” (3) are shaped by emotions and bodily states, and (4) are marked by intention and agency. The first – conscious percepts are differentiated and integrated – is also at the heart of Integrated Information Theory (IIT) (Tononi et al., 2016), where it is one of the “axioms” the theory departs from. This property seems to be fully explained by the dichotomy in functions described here, where the transition from unconscious to conscious vision is accompanied by the transition of functions that merely signal various low and high level features, to functions that enable these features to get integrated into a unitary and integrated whole. An example is the perception of a face. While face selective cells in IT cortex signal the category “face” (as opposed to say an animal or house), this is by no means similar to consciously seeing a face. In the conscious face percept, all features of that face (its color, shape, depth, motion, etc.) are integrated with the “faceness” of it (Fahrenfort et al., 2012). In Recurrent Processing Theory (RPT), the unconscious functions of feature extraction and categorizations are mediated by the feedforward sweep, while conscious functions related to perceptual organization are mediated by recurrent (feedback, or re-entrant) cortico-cortical connections (Lamme, 2010). It is not unlikely that experiencing some emotion to go along with that face also depends on recurrent interactions between neurons representing the different features of the face with some neurons signaling emotional content, residing either in subcortical structures like the amygdala of in ventromedial prefrontal cortices (Klasen et al., 2014).

Some argue that a mere visual representation, however integrated and organized, is not sufficient for having a conscious percept of it. Either some re-representation of it is deemed necessary, as in Higher Order Thought Theory (HOTT) (Brown et al., 2019), or functions related to access, such as attention and reportability, as in Global (Neuronal) Workspace Theory (GNWT) (Naccache, 2018). Although this may be necessary to report, memorize or otherwise cognitively manipulate the visual information, it remains to be demonstrated how such functions provide an insight into the transition from unconscious to conscious vision (Tsuchiya et al., 2015). Some integration is offered in the GNWT account, as upon entering the global workspace visual information may get integrated with information from other senses, emotions, or motor plans (Naccache, 2018). However, this is a type of integration operating at a higher level than the integration that seems to mark the transition form unconscious to conscious seeing that is discussed here. HOTT offers no explanation of the “integration” and “unity” aspect of consciousness, as the operations underlying this are fully completed before higher order thoughts are elevating the first order representation to consciousness (Brown et al., 2019). Both GNWT and HOTT may speak to the aspect of intention and agency, as indeed conscious visual percepts often go along with some sort of intention (for example to grasp). Given that vision can also be entirely passive, and is not lost in patients with even severe disturbance of agency (Bayne et al., 2019), I have a hard time seeing the explanatory power of these functions toward understanding seeing (as opposed to knowing, wanting, remembering, etc.).

Perhaps the most difficult aspect of conscious experience is that of it having this ego-perspective, or “I”-point of view. This is primarily caused by the “I” being hard to define, let alone pin down to some specific functions or neural processes. In some versions of HOTT, the re-representation of visual information is thought to be a pointer indicating whether the visual representation is coming from within (as in imagination) or from the outside (as in perception) (Brown et al., 2019). It is also conceivable that the process of perceptual organization generates the ego-perspective that vision has. For example, it was recently proposed (in the context of IIT) that the perception of space, as it unfolds in front of our eyes, is supported by brain areas whose units are linked by a grid-like connectivity, as in visual cortex (Haun and Tononi, 2019). Others link the ego-perspective to integrating visual information with interoceptive information (Park and Tallon-Baudry, 2014). Either way, also this aspect of consciousness seems quite distant from explaining the unconscious to conscious transition that is dealt with here, as both unconscious and conscious vision share the same “I,” being either aware of an object or not.

In sum, therefore, the transition from unconscious to conscious vision, key to understanding the NCC of seeing, is probably best explained by visual processing going from (low and high level) feature extraction toward integrating those features into a coherent and integrated whole, thereby constituting the organized and unitary percept that sits so prominently in the mind of the visual animal we are.

## Author Contributions

The author confirms being the sole contributor of this work and has approved it for publication.

### Conflict of Interest

The author declares that the research was conducted in the absence of any commercial or financial relationships that could be construed as a potential conflict of interest.

## Popular Summary

How is it possible that we have a conscious sensation of what we see? Several theories argue for higher level cognitive functions, such as attention, access or metacognition to play an important role. Here, I take a closer look at the visual functions that mark the transition from unconscious to conscious vision. It appears that a clear distinction between such functions can be made, hinting toward an important role for perceptual organization as the “machine” that generates the conscious visual percept.
